# RACE AND RURAL-URBAN DIFFERENCES IN HOME HEALTHCARE LATENCY IN PATIENTS WITH DEMENTIA

**DOI:** 10.1093/geroni/igac059.1543

**Published:** 2022-12-20

**Authors:** Amit Kumar

**Affiliations:** Northern Arizona University, Flagstaff, Arizona, United States

## Abstract

Given the need to ensure equity of timely access to post-acute care among minority groups in rural areas, there is a pressing need to understand disparities in the start of home healthcare among minorities with Alzheimer's disease and Related Dementia (ADRD). This study investigates differences in the timing of initiation of home healthcare services following acute hospitalization for ADRD patients by race/ethnicity and rural and urban locations. A secondary analysis was conducted among older adults with ADRD using Medicare data, discharged to home following an episode of acute hospitalization in 2016-2017. The study outcome was a delay in the start of home healthcare after two days of hospital discharge, defined as a home health latency. Compared to non-Hispanic Whites residing in urban areas, Blacks living in urban areas and Hispanics living in rural areas have a significantly higher odd of home health latency 1.15 (95%CI;1.11-1.18) and 1.06 (95%CI;1.02-1.10), respectively.

